# Effects of Acute Prolonged Sitting and Interrupting Prolonged Sitting on Heart Rate Variability and Heart Rate in Adults: A Meta-Analysis

**DOI:** 10.3389/fphys.2021.664628

**Published:** 2021-05-03

**Authors:** Lauren C. Bates, Abdullah Alansare, Bethany Barone Gibbs, Erik D. Hanson, Lee Stoner

**Affiliations:** ^1^Department of Exercise and Sport, University of North Carolina at Chapel Hill, Chapel Hill, NC, United States; ^2^Department of Health and Human Development, University of Pittsburgh, Pittsburgh, PA, United States; ^3^Department of Exercise Physiology, King Saud University, Riyadh, Saudi Arabia

**Keywords:** heart rate, heart rate variability, prolonged sitting, interrupting prolonged sitting, cardioautonomic function

## Abstract

Prolonged sitting increases cardiovascular disease (CVD) risk, however the physiological mechanisms contributing to CVD from acute sitting exposure are not well-understood. Therefore, this study investigated the heart rate (HR) and variability (HRV) responses to prolonged sitting and after interrupting prolonged sitting (e.g., walking). Electronic databases were searched (inception-August 2020) for studies which exposed adults to prolonged (≥1 h) sitting with and/or without interruptions. Twenty-one articles (27 trials, *n* = *537*) met inclusion criteria. Prolonged sitting non-significantly increased HR (weighted mean difference (WMD) = 0 bpm, 95% CI: −2, 3) and HRV (standardized mean difference (SMD) = 0.12, 95% CI: −0.08, 0.33) compared to pre-sitting baseline. Interrupting prolonged sitting yielded a non-significant small increase in HR (WMD = 4 bpm, 95% CI: 0, 7) compared to pre-sitting baseline. Sub-group analyses investigating interrupting prolonged sitting revealed small-to-moderate increases in HR in healthy populations (WMD = 6 bpm, 95% CI: 1, 10) and following walking interruptions (WMD = 7 bpm, 95% CI: 3, 11). In conclusion, prolonged sitting does not significantly affect HR or HRV. However, interrupting prolonged sitting yielded a small non-significant increase in HR, potentially indicative of increased metabolic demand. Further research is needed to investigate poor CVD outcomes via autonomic disruption from prolonged sitting.

## Introduction

Regular sedentary behavior exposure, defined as low energy expenditure (≤1.5 metabolic equivalents) in a seated, reclined, or lying posture (Tremblay et al., 2017), is associated with increased cardiovascular disease (CVD) incidence and mortality (Biswas et al., 2015). However, the mechanisms connecting acute prolonged sitting exposure to CVD risk are not well-understood. In order to understand how repeated exposure to prolonged sitting contributes to CVD overtime, we need to first understand the physiological response to a single bout of sitting. A recent meta-analysis (Paterson et al., 2020) reported that exposure to uninterrupted prolonged sitting leads to acute lower-extremity endothelial dysfunction, perhaps indicating a direct hemodynamic effect on the lower extremity vasculature. These effects may result from systemic cardiovascular effects where prolonged sitting causes blood pooling in the lower extremities (Dempsey et al., 2018), leading to reduced venous return, decreased stroke volume, and a subsequent decrease in lower extremity blood flow (Thosar et al., 2012; De Brito et al., 2015; Credeur et al., 2019). The decrease in lower extremity blood flow leads to a decrease in shear stress – the principal stimulus maintaining endothelial cell health (Wheeler et al., 2019). Additionally, the decrease in stroke volume can unload the baroreceptors, triggering the autonomic nervous system to increase heart rate (HR) in an attempt to maintain cardiac output while vasoconstricting resistance vessels to maintain blood pressure (Shaffer et al., 2014). Overtime, this may result in an elevated resting heart rate, which is associated with CVD risk (Altenburg et al., 2019). Furthermore, reduced heart rate variability (HRV) at rest is indicative of autonomic dysfunction, a known risk factor of CVD (Ebara et al., 2008), however research evaluating the effects of prolonged sitting exposure on cardio-autonomic function is limited and inconsistent in both findings and methodology (sitting duration, sitting control, measurement devices, etc.).

Breaking up prolonged sitting bouts with interruption strategies reduces blood pressure (Barone Gibbs et al., 2017a) and preserves lower extremity endothelial function (Paterson et al., 2020). Thus, interruption may help to protect the cardiovascular system potentially reducing the burden of blood pooling (decreasing resting HR) stimulating cardio-autonomic activity (increasing HRV). At present specific guidelines for interrupting sitting behavior, i.e., what to substitute prolonged sitting behavior with, are lacking. Interruption strategies including standing (Wennberg et al., 2016; Barone Gibbs et al., 2017b; Carter et al., 2018; Kruse et al., 2018; Dogra et al., 2019), walking (De Brito et al., 2015; Evans et al., 2019; Kowalsky et al., 2019), cycling (Sperlich et al., 2018; Dogra et al., 2019), calf-raises (Cabak et al., 2017), or strength training (Garten et al., 2018; Headid et al., 2020) have been tested. However, the findings have been inconsistent and the optimal strategy for interrupting prolonged sitting remains unknown. Summarizing the effects of sitting interruptions on the cardio-autonomic system can help to quantify effects, identify strengths and limitations of the existing literature, and indicate further research requirements.

### Objective

The purpose of this meta-analysis was to consolidate the literature investigating the effects of acute prolonged sitting on the cardio-autonomic system, specifically the HR and heart rate variability (HRV) responses (Shvartz et al., 1983; Moher et al., 2009; Higgins et al., 2011, 2019; Thosar et al., 2012; De Brito et al., 2015; Restaino et al., 2015; Wennberg et al., 2016; Barone Gibbs et al., 2017b; Cabak et al., 2017; Kerr et al., 2017; Vranish et al., 2017; Carter et al., 2018; Garten et al., 2018; Horiuchi et al., 2018; Kruse et al., 2018; Sperlich et al., 2018; Credeur et al., 2019; Dogra et al., 2019; Evans et al., 2019; Kowalsky et al., 2019; Headid et al., 2020). Additionally, a secondary purpose of this meta-analysis was to increase our understanding with regard to how interrupting sitting may attenuate CVD effects of prolonged sitting such as HR when available.

## Methods

This meta-analysis was carried out in accordance with PRISMA (Preferred Reporting Items for Systematic Reviews and Meta-Analyses) guidelines (Barendregt and Doi, 2015) and registered with PROSPERO (International Prospective Register of Systematic Reviews: CRD42020196685).

### Data Sources and Searches

Electronic databases (PubMed and Google Scholar) were searched by two authors (LB, AA) utilizing the following search terms: (sitting or sedentarism or sedentary or television time or screen time) AND (HRV or heart rate variability or heart rate).

The reference lists of all identified trials and relevant reviews or editorials were also examined. The search was limited titles and abstracts written in English language with human adult (18 years of age and older) populations published between database inception and August 31st, 2020.

### Article Selection

Two researchers (LB and AA) completed the study selection independently. For the purpose of this meta-analysis, the term “article” is used synonymously with “study” and “trial” is the unit included in the meta-analysis. A given article may have resulted in more than one eligible “trial” if the article included more than one HR and HRV measure and/or more than one intervention group or condition. Initially, article titles and abstracts were screened for relevance by two reviewers (LB and AA) and a third reviewer was used to settle a potential split-decision (LS). The full-text versions of potentially eligible articles were obtained to evaluate for eligibility, also by two reviewers (LB and AA). The following criteria were used to select trials for inclusion in this review: (i) English language, (ii) human studies, (iii) adults (18 years of age and older), (iv) inclusion of an uninterrupted prolonged (>1 h) sitting bout. The following criteria were used to select trials for the secondary objective of interrupting prolonged sitting (i) prolonged sitting occurred prior to interruption, (ii) subjects returned to sitting following interruption. Repeated publications from the same studies were excluded.

### Data Extraction and Quality Assessment

Data extraction, quality assessment, and investigation of the sitting interruption were completed by two reviewers (LB and LS). Data extracted for each eligible trial included bibliographic information (author, publication year), baseline participant characteristics, details of prolonged sitting bout (and interruption condition, if relevant), and reported outcomes.

Study quality was assessed using the RoB2: revised Cochrane risk-of-bias tool for randomized trials (range 1–3), which includes items related to randomization, deviations from intended interventions, missing data, measurement of the outcome, selection of the reported result, and overall bias (Assink and Wibbelink, 2016). Because it is difficult (if not impossible) to blind participants to a bout of prolonged sitting (or interruption to prolonged sitting), we considered blinding of the operator assessing the outcome as the quality criterion for this item. Additionally, pre-assessment guidelines (including abstaining from alcohol, tobacco, caffeine, food, and exercise) as well as quality of prolonged sitting (i.e., were bathroom breaks allowed and/or standardized) were further quality criterion.

### Data Synthesis

Aggregation (LB) and calculation of final results was conducted by one author (LS). For each outcome of interest, the pre- and post-prolonged sitting bout values (mean and standard deviation) were extracted. When mean differences and associated standard deviations were not published, they were estimated from the pre- and post-intervention values based on methods (calculated from standard errors or from confidence intervals) from the Cochrane Handbook for Systematic Reviews of Interventions (Doi et al., 2015a) or provided by the authors following contact. For studies reporting multiple time points, only the final time point was used in analyses. Primary study outcomes were HR (bpm) and HRV (Root mean square of the successive differences [RMSSD], standard deviation of all NN intervals [SDNN], high frequency [HF], low frequency [LF], high frequency/low frequency ratio [HF/LF], and/or total variance [TV]).

### Data Analysis

A single author (LS) conducted all statistical analysis using the MetaXL software (Doi et al., 2015b), and ancillary analysis using the metafor-package (Cohen, 1988) for the R statistical environment (RKWard version 0.7.1). HR parameter estimates are expressed as both the weighted mean difference (WMD) and standardized mean difference (SMD, Cohen's). Considering HRV measures are expressed using different units and scales, HRV parameter estimates are only reported as SMD. Data were pooled using the inverse variance heterogeneity (IVhet) model of meta-analysis to account for potential heterogeneity within and between studies (Sterne et al., 2011). This method has been shown to be a preferred alternative to the more traditional random-effects model which has been suggested to underestimate statistical error and produce overconfident estimates when using heterogenous data. Additionally, the IVhet models were adjusted according to the adjudicated quality (RoB2 score) of each included study (Duval and Tweedie, 2000). The SMD was used to assess the magnitude of effect, where <0.2, 0.2, 0.5, and 0.8 was defined as trivial, small, moderate, and large, respectively (Furuya-Kanamori et al., 2018).

Subsequent to running the IVhet and Quality Effects models, we examined the robustness of the pooled results and the potential for publication bias. Sensitivity analyses were conducted by removing one trial at a time. Measures of publication bias included visual inspection of Begg's funnel plots (Higgins et al., 2003), recalculation of the pooled estimate using Duval and Tweedie's trim and fill method (Cheung, 2019), and inspection of Doi plots in tandem with The Luis Furuya Kanamori (LFK) index (Sakowski et al., 2011). Doi plots are reported to be more objective than the qualitative Begg's funnel plot, and the LFK index has been reported to be a more sensitive quantitative measure of asymmetry and potential bias than the Egger's regression intercept test (Moher et al., 2009). Using the LFK method, <1 indicates no asymmetry, 1–2 suggests minor asymmetry, and >2 indicates major asymmetry (Moher et al., 2009). Last, statistical heterogeneity was assessed using the *I*^2^ statistic, where <25, 25–75, and >75% represent low, moderate, and considerable heterogeneity, respectively (Peddie et al., 2013). Heterogeneity >25% was assumed to indicate that effect sizes could not be treated as estimates of one common effect size, justifying *a priori* determined sub-group analysis.

The metafor-package for R statistical software was used to conduct ancillary analyses (Crespo et al., 2016). Meta-analysis statistical models typically assume independent effect sizes (Crespo et al., 2016). For the current meta-analysis, most included studies reported more than one estimate of HRV (e.g., RMSSD and HF). To more robustly account for effect size dependency, a 3-level model was conducted with restricted maximum likelihood estimation (Cohen, 1988; Crespo et al., 2016). The 3 sources of variance taken into account included: variance at the level of the subject (Level 1), variance between effect sizes extracted from the same study (Level 2), and variance between studies (Level 3). To determine the significance of the level 2 and level 3 variance, the full model was compared to a model excluding one of these variance parameters in two separate log-likelihood ratio tests. In the event of significant level- 2 and/or level-3 variance, the distribution of effect sizes was considered heterogeneous.

## Results

### Literature Search and Trial Selection

**Figure 1:**
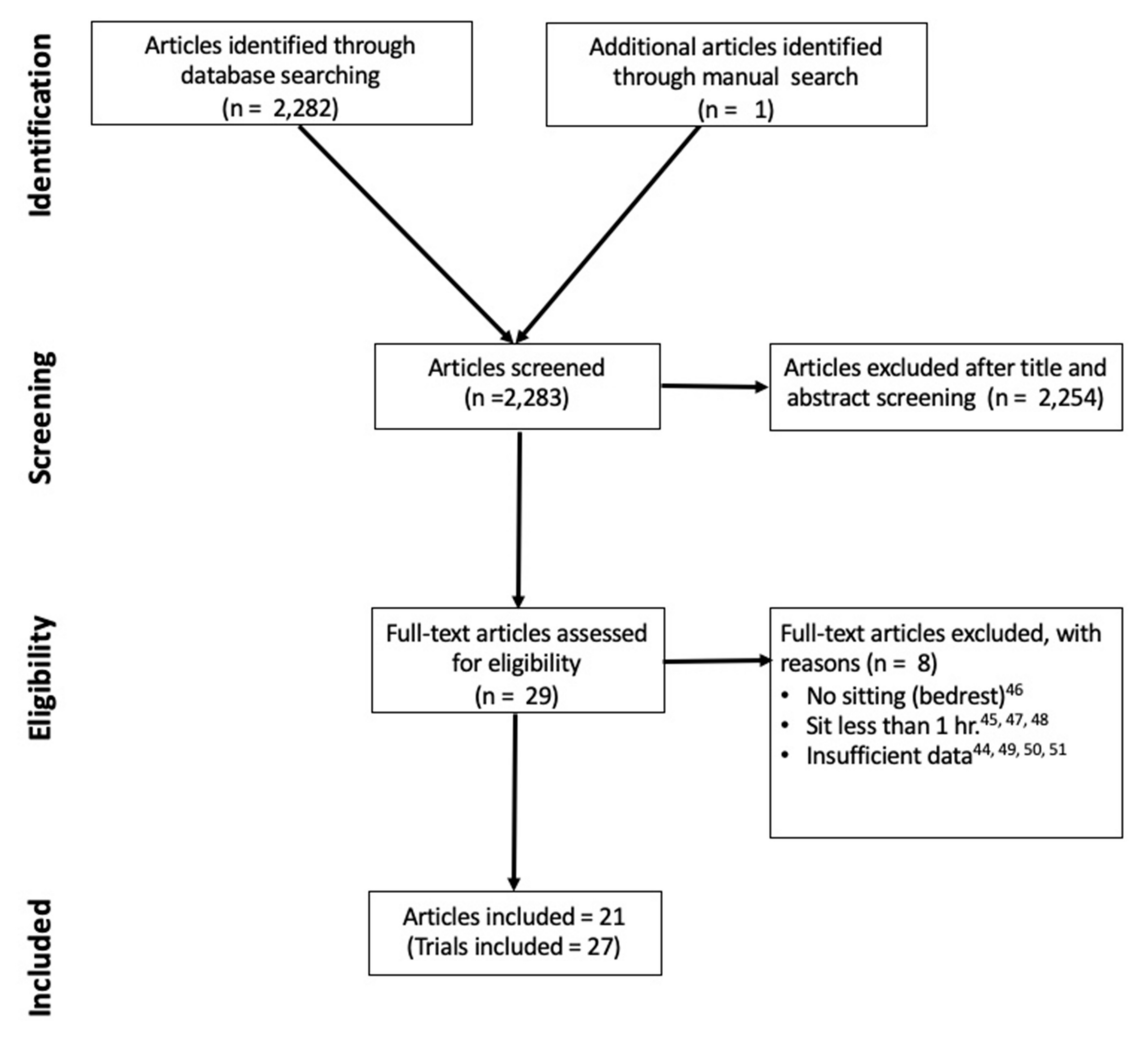
Flow chart including inclusion and exclusion criteria. hr, hour.

Figure 1 outlines the literature search strategy. A total of 2,283 potentially eligible articles were identified. Following screening of titles and abstracts, 2,254 articles were excluded because they did not meet selection criteria. Of these, 29 randomized cross-over trials underwent full text screening and 8 were excluded (Chaput and Tremblay, 2007; Bourdin et al., 2010; Jean-François et al., 2010; Larsen et al., 2014; Restaino et al., 2015; Botter et al., 2016; Garten et al., 2018; Snarr et al., 2019). The final analysis included 22 studies (27 trials) with 21 studies reporting HR data (Shvartz et al., 1983; Chowienczyk et al., 1994; Higgins et al., 2011, 2019; Thosar et al., 2012; De Brito et al., 2015; Restaino et al., 2015; Wennberg et al., 2016; Barone Gibbs et al., 2017b; Cabak et al., 2017; Vranish et al., 2017; Carter et al., 2018; Garten et al., 2018; Horiuchi et al., 2018; Sperlich et al., 2018; Credeur et al., 2019; Dogra et al., 2019; Evans et al., 2019; Kowalsky et al., 2019; Cheng-Hsuan et al., 2020; Headid et al., 2020) and seven studies reporting HRV data (Shvartz et al., 1983; Chowienczyk et al., 1994; Thosar et al., 2012; Restaino et al., 2015; Cabak et al., 2017; Kruse et al., 2018; Credeur et al., 2019). Additionally, 13 of the HR studies reported interruption conditions to the bout of prolonged sitting (De Brito et al., 2015; Wennberg et al., 2016; Barone Gibbs et al., 2017b; Cabak et al., 2017; Vranish et al., 2017; Carter et al., 2018; Garten et al., 2018; Kruse et al., 2018; Sperlich et al., 2018; Dogra et al., 2019; Evans et al., 2019; Kowalsky et al., 2019; Headid et al., 2020), however there were not enough HRV studies reporting interruption conditions (Cabak et al., 2017; Kruse et al., 2018) to summarize with meta-analysis.

### Description of the Included Trials

#### Trial Setting and Participants

Characteristics of included trials are summarized in Table 1. The number of participants in each trial ranged from 8 (Higgins et al., 2011) to 124 (Horiuchi et al., 2018). One HR trial included only females (Vranish et al., 2017) and 4 HR trials (Higgins et al., 2011; Barone Gibbs et al., 2017b; Credeur et al., 2019; Cheng-Hsuan et al., 2020) and 1 HRV (Credeur et al., 2019) trial included only males. The mean age of the participants ranged from 19 ± 0 (Cheng-Hsuan et al., 2020) to 67 ± 7 years (Garten et al., 2018). Seven trials included clinical populations with cardiometabolic diseases including one trial with hypertensive participants (Credeur et al., 2019), four trials with overweight/obese participants (Vranish et al., 2017; Garten et al., 2018; Dogra et al., 2019; Kowalsky et al., 2019), and two trials with both hypertension and overweight/obese participants (Wennberg et al., 2016; Carter et al., 2018). Bouts of prolonged sitting ranged from 1 (Headid et al., 2020) to 9 (Dogra et al., 2019) h with an average duration of 5 h. It should also be noted that all HRV studies (Shvartz et al., 1983; De Brito et al., 2015; Vranish et al., 2017; Carter et al., 2018; Garten et al., 2018; Wheeler et al., 2019) *l* for <3 h, which was characterized as a short bout of sitting. Of the 22 trials, 13 included interruptions to sitting (De Brito et al., 2015; Wennberg et al., 2016; Barone Gibbs et al., 2017b; Cabak et al., 2017; Vranish et al., 2017; Carter et al., 2018; Garten et al., 2018; Kruse et al., 2018; Sperlich et al., 2018; Dogra et al., 2019; Evans et al., 2019; Kowalsky et al., 2019; Headid et al., 2020). The duration and frequency of interruption varied in length from 10 calf raises every 10 min (Cabak et al., 2017) to 30 min of walking once (De Brito et al., 2015). The trials included varying modalities of interruption strategies as well including standing, walking, biking and/or pedaling, calf-raises, or strength training interruptions.

**Table 1:** Characteristics of included trials.

	**References**	**Quality**	**Sample [*n* (f); mean age, years (SD)]**	**Disease**	**HR**	**HRV**	**Sit Duration (hours)**	**Frequency**	**Time**	**Type**
Interruption	Altenburg et al. (2019)	2	20 (0); 19 (0)	N/A	yes	no	5	1 × 1 h	10 min	Stand
	Carter et al. (2018)	3	15 (5); 36 (10)	N/A	yes	no	4	1 × 30 min	2/8 min	Walk
	Dogra et al. (2019)	2	10 (5); 25 (3)	N/A	yes	no	4	1 × 1 h	3 min	Bike
	Ebara et al. (2008)	2	24 (12); 21 (1)	N/A	no	yes	2.5	1 × 10 min	5 min	Stand
	Evans et al. (2019)	2	20 (14); 22 (3)	N/A	yes	yes	3	1 × 10 min	10 reps	Calf Raise
	Barone Gibbs et al. (2017b)	3	18 (9); 39 (13)	N/A	yes	no	8	1 × 30 min	30 min	Stand
	Barone Gibbs et al. (2017a)	2	25 (9); 42 (12)	OW + HTN	yes	no	1	1 × 30 min	30 min	Stand
	Kerr et al. (2017)	2	10 (10); 66 (9)	OW	yes	no	5	1 × 20 min/1 × 60 min	2/10/10 min	Stand/walk/stand
	Kowalsky et al. (2019)	3	14 (12); 53 (10)	OW	yes	no	4	1 × 1 h	2 set × 15 reps	RT
	Kruse et al. (2018)	2	13 (3); 38 (3)	OW	yes	no	6	1 × 1 h	10 min	Stand/Bike
	Sperlich et al. (2018)	2	12 (7); 22 (2)	N/A	yes	no	3	1 × 1 h	6 min	HIIT
	Wennberg et al. (2016)	2	19 (9); 60 (8)	OW	yes	no	5	1 × 30 min	3 min	Walk
	Wheeler et al. (2019)	2	67 (35); 67 (7)	N/A	yes	no	8	1 × 1 h	30 min	Walk
Sit-Only										
	Cabak et al. (2017)	3	124 (55); 34 (8)	N/A	yes	no	8	-	-	-
	Credeur et al. (2019)	2	20 (7); 26 (7)	N/A	yes	yes	3	-	-	-
	De Brito et al. (2015)	2	16 (0); 32 (7)	HTN	yes	yes	3	-	-	-
	Garten et al. (2018)	2	20 (2); 25 (1)	N/A	yes	yes	3	-	-	-
	Headid et al. (2020)	2	23 (6); 22 (2)	N/A	yes	yes	2.5	-	-	-
	Horiuchi et al. (2018)	3	18 (6); 21 (1)	N/A	yes	yes	3	-	-	-
	Kerr et al. (2017)	2	10 (10); 66 (9)	OW	yes	no	5	-	-	-
	Restaino et al. (2015)	2	11 (0); 27 (1)	N/A	yes	no	6	-	-	-
	Shvartz et al. (1983)	2	8 (0); 25 (0)	N/A	yes	no	3	-	-	-
	Vranish et al. (2017)	2	20 (12); 21 (1)	N/A	yes	no	8	-	-	-

*Study quality was assessed using the RoB2: revised Cochrane risk-of-bias tool for randomized trials (range 1–3). f, female; SD, standard deviation; F, frequency; T, time; T, type; OW, overweight; OW+HTN, overweight and hypertensive; HTN, hypertensive; HR, heart rate; HRV, heart rate variability; RT, resistance training; HIIT, high intensity interval training*.

### Methodological Quality Assessment

As reported in Table 1, the quality of the included studies ranged from 1 to 3 out of 3. Overall, included studies had a median quality score of 2 indicating fair quality.

### Synthesis of the Results

#### Prolonged Sitting: Heart Rate

**Figure 2:**
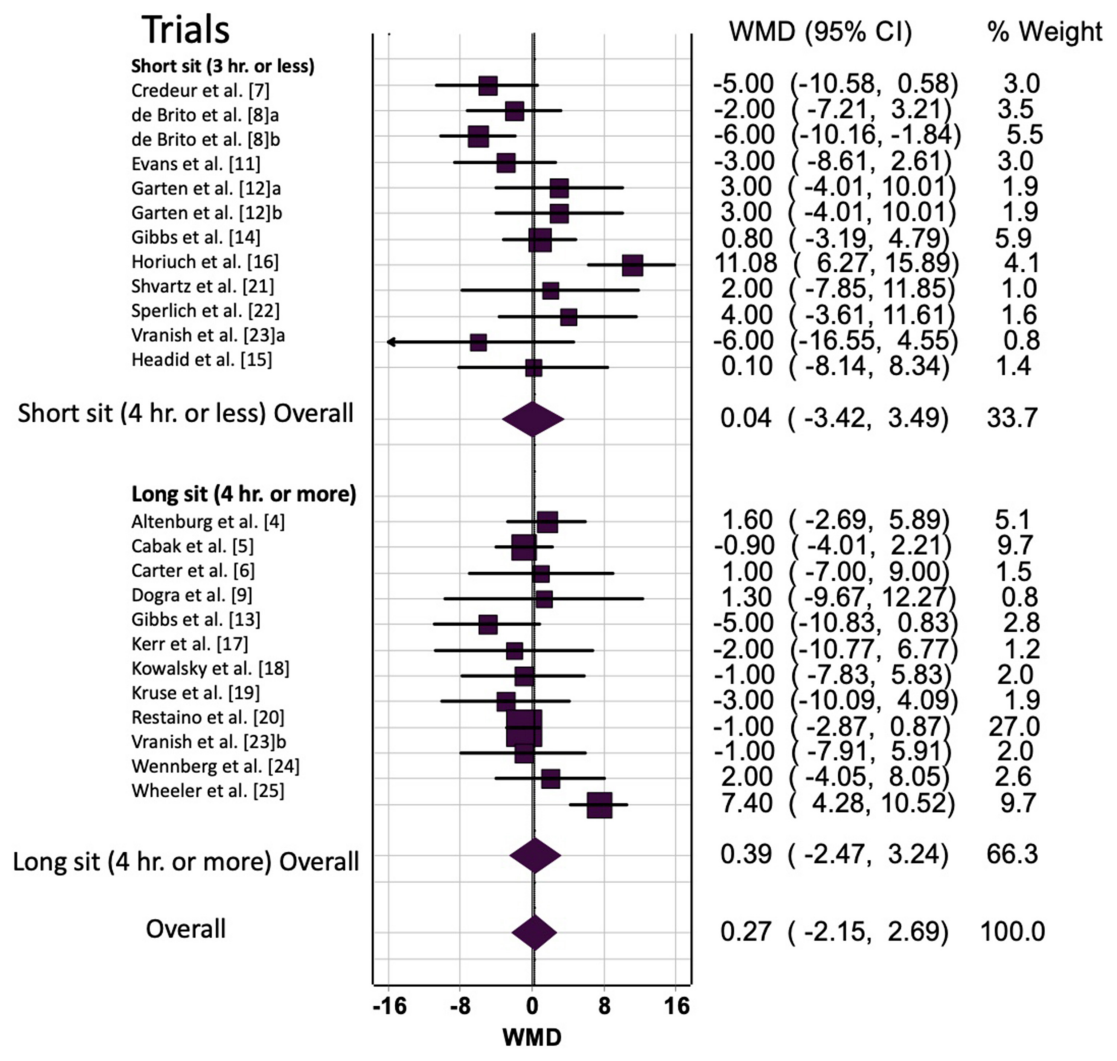
Prolonged sitting bout duration (short vs. long) effect on heart rate. a/b, multiple trials within the same study; hr, hours; WMD, weighted mean difference; BPM, beats per minute; CI, confidence interval.

Prolonged sitting resulted in a trivial, non-significant increase in HR (Table 2). Sensitivity analysis indicated that none of the trials unduly influenced the pooled estimate. Visual inspection of the funnel plot did not reveal asymmetry (Supplementary Figure 2) and the estimate does not change despite almost reaching significance. Additionally, the LFK index was 0.649 and indicated no asymmetry. The heterogeneity was moderate (*I*^2^ = 65%, *p* < 0.001), which may be explained partially by sample population differences. However, sub-group analysis did not reveal any effects of age, sex, cardiometabolic status (CMD), or sitting duration on the pooled estimate (Figure 2).

**Table 2:** The effect of prolonged sitting on heart rate.

	**Trials**	**Sample**	**Quality**	**Pooled effect**					**Asymmetry**	**Heterogeneity**		
	**(*n*)**	**(*n*)**	**/3**	**WMD**	**LCI**	**UCI**	**SMD**	***P***	**LFK**	**Q**	***P***	***I*^**2**^**
**All**	24	285	2.2	0	−2	3	0.05	0.827	0.649	64.9	<0.001	65
**Age**												
<40 years	18	355	2.2	0	−3	2	−0.05	1.000		39.2	<0.001	57
>40 years	6	153	2.3	3	−2	7	0.28	0.278		18.6	<0.001	19
**Sex**												
Men	5	71	2.0	−1	−4	3	−0.25	1.000		7.25	0.120	45
Women	1	10	2.0	−2	−11	67	−0.20	1.000				
Mixed	18	427	2.3	2	−1	4	0.10	0.271		49.3	<0.001	66
**CMD status**												
Healthy	16	377	2.2	1	−3	4	0.11	0.735		59.1	<0.001	73
Diseased	8	131	2.6	−1	−3	1.12	−0.15	1.000		4.03	<0.001	65
**Sitting duration**												
<4 h	11	160	2	0	−3	3.49	−0.02	0.986		37.2	<0.001	70
>4 h	13	348	2	0	−2	3.24	0.08	0.802		27.6	<0.001	60

*SMD: trivial, small, moderate and large effect sizes are defined as <0.2, 0.2, 0.5, and 0.8, respectively. LFK: <1 indicates no asymmetry, 1–2 suggests minor asymmetry, and >2 indicates major asymmetry. I^2^: 25, 50, and 75% represent low, moderate, and high heterogeneity, respectively. WMD, weighted mean difference; LCI, lower confidence interval; UCI, upper confidence interval; SMD, standardized mean difference; LFK, Luis Furuya-Kanamori Index; CMD, cardiometabolic disease*.

#### Prolonged Sitting With Interruption: Heart Rate

Overall and sub-group analyses are reported in Table 3 and Figure 2 displays the forest plot for trials grouped by <4 or >4 h. Across all trials prolonged sitting with interruption resulted in a small non-significant increase in HR (WMD = 4 bpm, 95% CI: 0, 7, *p* = 0.02). Sensitivity analysis indicated that none of the trials unduly influenced the pooled estimate. Visual inspection of the funnel plot revealed asymmetry (Supplementary Figure 2), which was supported by the LFK index −2.78 of (major asymmetry). The trim and fill analysis decreased the WMD to 2 bpm (95% CI: 0, 5) (Supplementary Figure 2). Additionally, the heterogeneity was considerable (*I*^2^ = 73%, *p* < 0.001), which may be explained by variation in the strategy for interrupting prolonged sitting, age or biological sex differences, or prolonged sitting duration variation between studies.

**Table 3:** The effect of prolonged sitting with interruption on HR.

	**Trials**	**Sample**	**Quality**	**Pooled effect**					**Asymmetry**	**Heterogeneity**		
	**(*n*)**	**(*n*)**	**/3**	**WMD**	**LCI**	**UCI**	**SMD**	***P***	**LFK**	**Q**	***P***	***I^**2**^***
**All**	17	358	2.2	4	0	7	0.38	0.052	−2.78	72.6	<0.001	78
**Age**												
<40 years	8	118	2.3	1	−3	4	0.03	0.717		15.3	0.030	54
>40 years	9	240	2.2	5	0	11	0.56	0.060		47.5	<0.001	83
**Sex**												
Men	1	20	2.0	1	−3	6	0.18	0.585				
Women	3	30	2.0	7	2	12	0.69	0.007				
Mixed	13	308	2.3	4	0	8	0.37	0.088		69.5	<0.001	83
**CMD status**												
Healthy	8	226	2.3	6	1	10	0.60	0.008		28.4	<0.001	75
Diseased	9	132	2.2	0	−5	4	0.01	1.000		28.1	<0.001	72
**Sitting duration**												
<4 h	3	40	2.0	−2	−13	9	−0.24	1.000		18.8	<0.001	89
>4 h	14	308	2.3	5	1	8	0.48	0.007		41.9	<0.001	69
**Modality**												
Stand	6	96	2.2	−1	−7	4	−0.14	1.000		19.4	<0.01	74
Walk	6	193	2.3	7	2	11	0.70	0.001		14.7	0.010	66
Other	5	69	2.2	3	−3	8	0.23	0.339		11.2	0.020	64

*SMD: trivial, small, moderate and large effect sizes are defined as <0.2, 0.2, 0.5, and 0.8, respectively. LFK: <1 indicates no asymmetry, 1–2 suggests minor asymmetry, and >2 indicates major asymmetry. I^2^: 25, 50, and 75% represent low, moderate, and high heterogeneity, respectively. WMD, weighted mean difference; LCI, lower confidence interval; UCI, upper confidence interval; SMD, standardized mean difference; LFK, Luis Furuya-Kanamori Index; CMD, cardiometabolic disease*.

When the populations were grouped by age, there was a non-significant moderate increase in HR for those aged >40 years (WMD = 5 bpm, SMD = 0.56, *p* = 0.060) and a negligible change for those aged under <40 years (WMD = 1 bpm, SMD = 0.03, *p* = 0.717). For women, there was a moderate increase in HR (WMD =7 bpm, SMD = 0.69, *p* = 0.007) but non-significant change for men (*p* = 0.585) and mixed sex (*p* = 0.088). For populations without CMDs, there was a moderate increase in HR (WMD = 6 bpm, SMD = 0.60, *p* = 0.008), and, for populations with CMDs, there was a non-significant change (*p* = 1.000). When the sitting bout length was >4 h, there was a moderate increase in HR (WMD = 5 bpm, SMD = 0.48, *p* = 0.007), and when the bout was <4 h there was a small, non-significant (*p* = 1.000) change. Last, there was a moderate increase in HR for walking-based interruptions (WMD = 7 bpm, SMD = 0.70, *p* = 0.001), but non-significant changes for standing (*p* = 1.000) or other (*p* = 0.339) interruptions.

#### Prolonged Sitting: Heart Rate Variability

**Figure 3:**
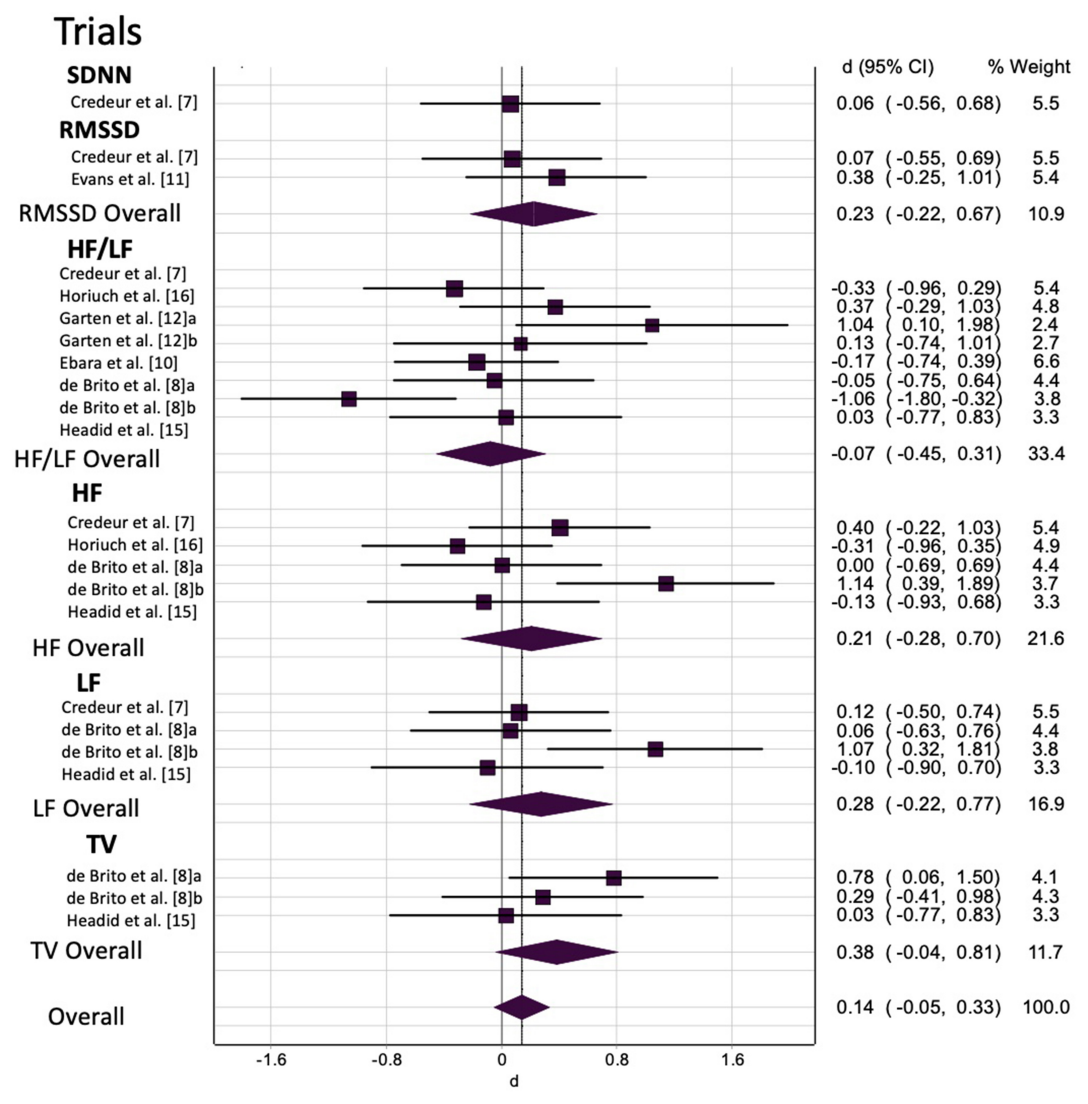
The effect of prolonged sitting on heart rate variability by outcome measure. a/b, multiple trials within the same study; SDNN, standard deviation of NN intervals; RMSSD, root mean of successive difference; HF, high frequency; LF, low frequency; HF/LF, high frequency/low frequency ratio; TV, total variance; WMD, weighted mean difference; CI, confidence interval.

Overall and sub-group analyses are reported in Table 4 and Figure 3 displays the forest plot for trails grouped by HRV outcome type. Prolonged sitting resulted in a trivial, non-significant change in HRV of SMD = 0.12 with *p* = 0.228. Sensitivity analysis indicated that none of the trials unduly influenced the pooled estimate. Visual inspection of the funnel plot did not reveal asymmetry, which was confirmed by the LFK index of 0.52. The was moderate heterogeneity (*I*^2^ = 50%, *p* < 0.001), which was potentially explained by the differences in biological sex differences or the use of different HRV outcomes. When the populations were grouped by sex for sub-group analysis, there were negligible-small, non-significant changes in HRV for men (SMD = 0.27, *p* = *0.287*) and mixed-sex (SMD = 0.06*, p* = *0.388*). When the trials were grouped by HRV outcome type, there were negligible-small and non-significant changes for out outcomes (SMD = 0.06–0.38, *p* = 0.076–1.000).

**Table 4:** The effect of prolonged sitting on HRV.

	**Trials**	**Sample**	**Quality**	**Pooled effect**				**Asymmetry**		**Heterogeneity**	
	**(n)**	**(n)**	**/3**	**SMD**	**LCI**	**UCI**	***P***	**LFK**	**Q**	***P***	***I*^**2**^**
**All**	23	376	2.3	0.12	−0.08	0.33	0.228	0.52	41.6	0.007	47
**Sex**											
Men	8	128	2.0	0.27	−0.22	0.75	0.287		25.6	<0.001	73
Mixed	15	248	2.4	0.08	−0.10	0.26	0.388		14.2	0.430	2
**Measure**											
SDNN	1	20	3.0	0.06	−0.56	0.68	0.855				
RMSSD	2	40	2.5	0.23	−0.22	0.67	0.321		0.00	0.500	0
HF/LF	8	126	2.3	−0.07	−0.45	0.31	1.000		15.0	0.040	53
HF	5	82	2.2	0.14	−0.45	0.72	0.658		13.5	0.010	70
LF	4	64	2.3	0.28	−0.22	0.77	0.278		5.79	0.120	48
TV	3	44	2.0	0.38	−0.04	0.81	0.076		1.98	0.370	0

*SMD: trivial, small, moderate and large effect sizes are defined as <0.2, 0.2, 0.5, and 0.8, respectively. LFK: <1 indicates no asymmetry, 1–2 suggests minor asymmetry, and >2 indicates major asymmetry. I^2^: 25, 50, and 75% represent low, moderate, and high heterogeneity, respectively. WMD, weighted mean difference; LCI, lower confidence interval; UCI, upper confidence interval; SMD, standardized mean difference; LFK, Luis Furuya-Kanamori Index; CMD, cardiometabolic disease*.

We attempted to account multiple HRV estimates (RMSSD, SDNN, HF, LF, HF/LF, TV) within a single trial by using two approaches: (i) sub-group analysis by HRV estimate (e.g., a given study may include both RMSSD and HF) as reported in the previous paragraph; and (ii) a multi-level meta-analysis model. The findings were consistent when we examined the data with the multi-level model, which revelated that all the heterogeneity was at level 1, suggesting our first approach was robust.

## Discussion

The aim of this meta-analysis was to synthesize the existing literature investigating the effects of acute exposure to prolonged sitting, with and without sitting interruptions, on HR and HRV. The main findings were that prolonged sitting without interruption does not significantly affect HR or HRV. Interrupting prolonged sitting with walking increased HR while standing or other (strength training, calf-raises, or biking) interruption modalities had trivial-small, non-significant effects, respectively. There were not enough HRV studies with an interruption condition to examine HRV response to interrupting prolonged sitting.

### Limitations

Several limitations should be borne in mind when considering these findings. First, the sample size of included trials was small, with a limited number of trials reporting HRV (*n* = 7). The trials that reported HRV utilized different HRV assessment methodology and reported a variety of outcomes, resulting in different units of frequency and time components. This was addressed by standardizing the unit of measure and by performing sub-group analysis by HRV assessment type (i.e., SDNN, RMSSD, HF/LF, HF, LF, or TV) and conducting a multi-level analysis. Second, the small number of HRV trials limits the conclusions drawn from the sub-group analysis. We were unable to investigate the effect of interrupting prolonged sitting on HRV as only two HRV trials reported interruption conditions. It should be noted that we attempted to perform an additional sub-group analysis of age using study population recruitment criteria (for example: 25–65 years old) instead of average age because mean age includes a large age range. However, this was not possible as only a few studies reported recruitment criteria. Lastly, the majority of the HRV trials were mixed sex, and it remains unclear whether both sexes respond similarly.

### Uninterrupted Prolonged Sitting: HR and HRV

Overall, there was a non-significant effect of acute exposure to prolonged sitting on HR and HRV. We speculated that HR would increase in response to prolonged sitting, and that greater increases would be observed in older aged or CMD populations compared to young and/or healthy populations. Additionally, we believed that HRV would decrease following acute prolonged sitting, again with more pronounced adverse effects in older and CMD participants (compared to younger and healthy participants). Our hypothesized mechanism proposed that blood pooling in the lower extremities as a consequence of prolonged sitting (Dempsey et al., 2018) would then lead to a reduction in venous return (decrease in stroke volume and cardiac output) (Thosar et al., 2012; De Brito et al., 2015; Credeur et al., 2019). Baroreceptors would then signal on afferent pathways to cardiovascular centers in the medulla, triggering a decrease in parasympathetic activation along with an increase in sympathetic activation to the heart and vessels (Shaffer et al., 2014). Thus, following, we would expect an increase in HR and a decrease in HRV. By way of explanation for the lack of support for this effect in this meta-analysis, it is possible that the balance between parasympathetic and sympathetic activity is unchanged with acute prolonged sitting (Restaino et al., 2015) or that sympathetic stimulation may change independently to parasympathetic stimulation. Further studies are warranted which combined HRV with measures of SNS activity, perhaps including muscle sympathetic nerve activity, galvanic skin sensors, or pupil dilation, to explore these potentially complex cardio-autonomic responses.

### Interrupting Prolonged Sitting: HR

Prolonged sitting with interruption approached statistical significance and resulted in a small increase in HR (WMD = 4 bpm). An increase in HR with interruption was expected and represents an increased metabolic demand following muscle contractions. Sub-group analyses revealed moderate effects (increase) of sitting interruption on HR in healthy populations (*p* = 0.008), for females (*p* = 0.007), and when interrupting longer bouts of sitting (*p* = 0.007). Additionally, walking as the interruption strategy resulted in the greatest and only significant increase in HR (*p* = 0.001), as compared to standing or other strategies. Since this meta-analysis measured the change in HR from before and after the sitting bout with interruption conditions (and not immediately after the interruption), we can speculate that an increase in HR is a result of the cardio-autonomic system acclimating to the lack of increase in blood pooling, therefore preserving stroke volume in order to maintain venous return. The aforementioned mechanism may also explain the lack of effect of sitting interruptions on HR in populations with CMDs. That is, in populations with CMD, sitting interruptions may be ineffective at preventing blood pooling in the lower extremities – at least with the strategies that have been trialed thus far. Additionally, the increase in HR for female populations is potentially indictive of the protective effect of estrogen receptors in the endothelium. The 17 β-estradiol (E2) receptor increases the production of nitric oxide (vasodilator) in endothelium and preserve healthy endothelial function (Wheeler et al., 2019). The presence of E2 in women may be responsible for our ability to detect the increase in HR following interruption at the post-prolonged sitting bout measure because the physiologic effect (increase in HR) of the interruption may last longer in women due to superior endothelial function compared to men (Chowienczyk et al., 1994).

It should be acknowledged that HR and HRV do not provide a comprehensive insight into the influence of interrupting prolonged sitting on the cardio-autonomic system. Studies examining both parasympathetic and sympathetic nervous system activity are needed to better understand the role the autonomic nervous system plays in the mechanisms involved in uninterrupted prolonged sitting which contribute to CVD risk. Future studies should consider measuring muscle sympathetic nervous activity or use of galvanic skin sensors, as well as the use of rate pressure product to determine myocardial load, which would provide us with additional knowledge about whether the HR response is protective or not. Additionally, consideration should be given to heart rate rhythm complexity, which measures the complexity of the total cardiovascular system rather than a sub-component univariate variable (i.e., HRV) alone (Cheng-Hsuan et al., 2020). Finally, investigators should keep in mind that there was variation in frequency, duration, and type of interruption strategy as well as when HR was measured (i.e., before, after, or during interruption) in the current literature. Standardizing guidelines or direct, within-study comparisons of prolonged sitting interruption strategies may yield more meaningful results.

### Implications

This meta-analysis is the first to our knowledge to consolidate the existing literature in this area in hope to investigate potentially deleterious effects of prolonged sitting. Such investigations on this and other outcomes are crucial for informing sedentary behavior intervention strategies, including optimization of interruption (i.e., frequency, intensity, and timing). Our findings are summarized in Table 5. The data indicates that a prolonged sitting bout (>1 h) resulted in trivial and non-significant increases in HR and HRV. However, interrupting sitting resulted in a small increase in HR especially apparent in healthy female populations. Current data suggests walking may be the optimum sitting interruption strategy. In the future, prolonged sitting studies should include additional measures of the cardio-autonomic system in order to better understand autonomic balance.

**Table 5:** Summary.

**What did we know prior to this study?**
• Sedentary behavior, and especially prolonged sitting, is linked to cardiovascular disease incidence • Acute prolonged sitting bouts result in vascular dysfunction, a precursor to cardiovascular disease • Interrupting prolonged sitting may offset vascular dysfunction
**What did we not know prior to this study?**
• Studies of the effect of prolonged sitting on HR and HRV have reported inconsistent results • How interrupting prolonged sitting affects HR and HRV compared to continuous prolonged sitting
**What does this study add?**
• Prolonged sitting does not affect heart rate or heart rate variability • Interrupting prolonged sitting results in a small non-significant increase in heart rate • The effect of interrupting prolonged sitting on HRV remains unclear due to insufficient data
**How do we use this new information?**
• Future research should focus on including HRV and other measures of cardioautonomic function • Simple interruption strategies like walking could counteract the adverse effects of prolonged sitting by activating the muscle pump to redistribute blood pooled in the lower body or by increasing metabolic demand
**What needs to happen next to move the field forward?**
• Optimal interruption strategies to interrupt sitting to improve cardiovascular function remain unclear, however walking may have the most beneficial potential • Research allowing for direct comparison of interruption strategies either within-study or across studies with similar methodology is needed • Studies investigating acute prolonged sitting effects on heart rate and heart rate variability should directly compare populations with different age and CMD status

There are important gaps in the current literature which were identified. For example, due to the low number of included trails which investigated the effect of interrupting sitting on HRV, the current meta-analysis was unable to draw any firm conclusions. Additionally, the wide variety in sitting interruption strategies make it difficult to determine the optimal prescription for sedentary behavior guidelines. Thus, direct within-study comparisons of interruption strategies as well as standardized frequency, intensity, and duration guidelines for prolonged sitting interruptions across studies that facilitate comparisons may result in a better understanding of the cardio-autonomic mechanisms contributing to the CVD risk associated with sedentary behavior. It is also worth noting that, like exercise guidelines, sedentary behavior guidelines should consider whether specific guidelines are required for special populations, including those with CMDs or disabilities.

## Conclusion

Sedentary behaviors, like prolonged sitting, are associated with CVD incidence and mortality. This meta-analysis consolidated the existing literature on the effects of prolonged sitting, with and without interruption, on HR and HRV. Furthermore, gaps in the existing literature were identified in order to better inform investigators in establishing the optimum guidelines to mitigate prolonged sitting. The results of this analysis suggest that there are not meaningful changes in HR or HRV during uninterrupted sitting bouts (>1 h). Interrupting sitting resulted in a non-significant increase in HR, which may be indicative of the increase in metabolic demand. Future research is needed in order to optimize interruption strategies including frequency, duration, and intensity and more sitting interruption studies investigating HRV are needed to better inform sedentary behavior guidelines.

## Data Availability Statement

The raw data supporting the conclusions of this article will be made available by the authors, without undue reservation.

## Author Contributions

LB, AA, BG, and LS: conceptualization, methodology, and formal analysis and investigation. LB and LS: writing - original draft preparation. All authors: writing - review and editing.

## Conflict of Interest

The authors declare that the research was conducted in the absence of any commercial or financial relationships that could be construed as a potential conflict of interest.
