# Supplementary material for: Effects of Acute Prolonged Sitting and Interrupting Prolonged Sitting on Heart Rate Variability and Heart Rate in Adults: A Meta-Analysis
Source: Front Physiol. 2021 May 3;12:664628. doi: 10.3389/fphys.2021.664628 (PMC8126673; doi:10.3389/fphys.2021.664628)
Supplement: Supplementary file 1 [file Data_Sheet_1.docx]

Supplementary Material

# Supplementary Figures

**Supplementary Figure 1: Trim and Fill for Prolonged Sitting HR**


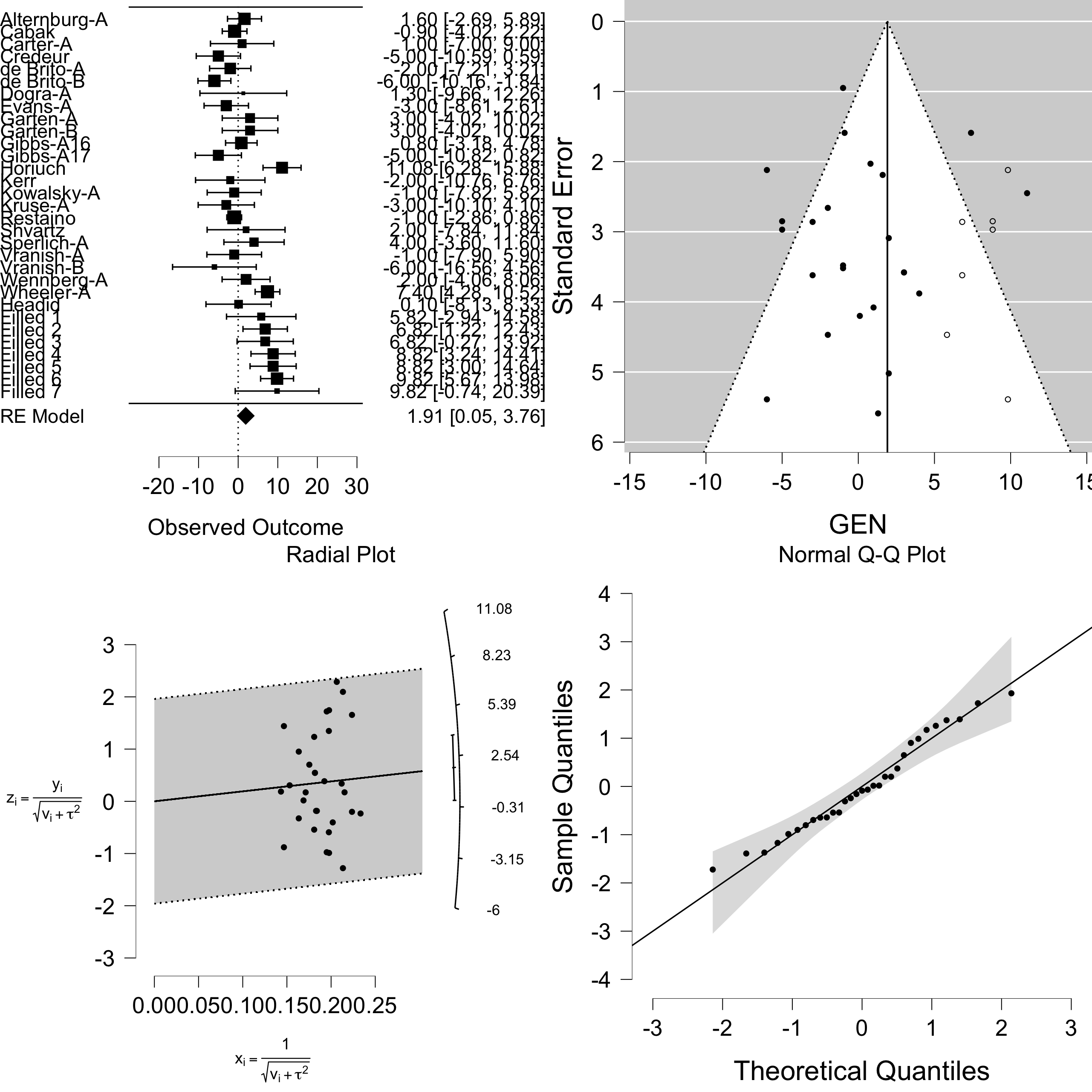


Interpretation: The original funnel plot revealed an overall effect of 0.27. After the trim and fill was performed, the overall effect increased to 1.91 indicating that publication bias exists. When hypothetical studies are added via the trim and fill, we see an increase in HR in response to prolonged sitting.

**Supplementary Figure 2: Trim and Fill for Interrupting Prolonged Sitting HR**


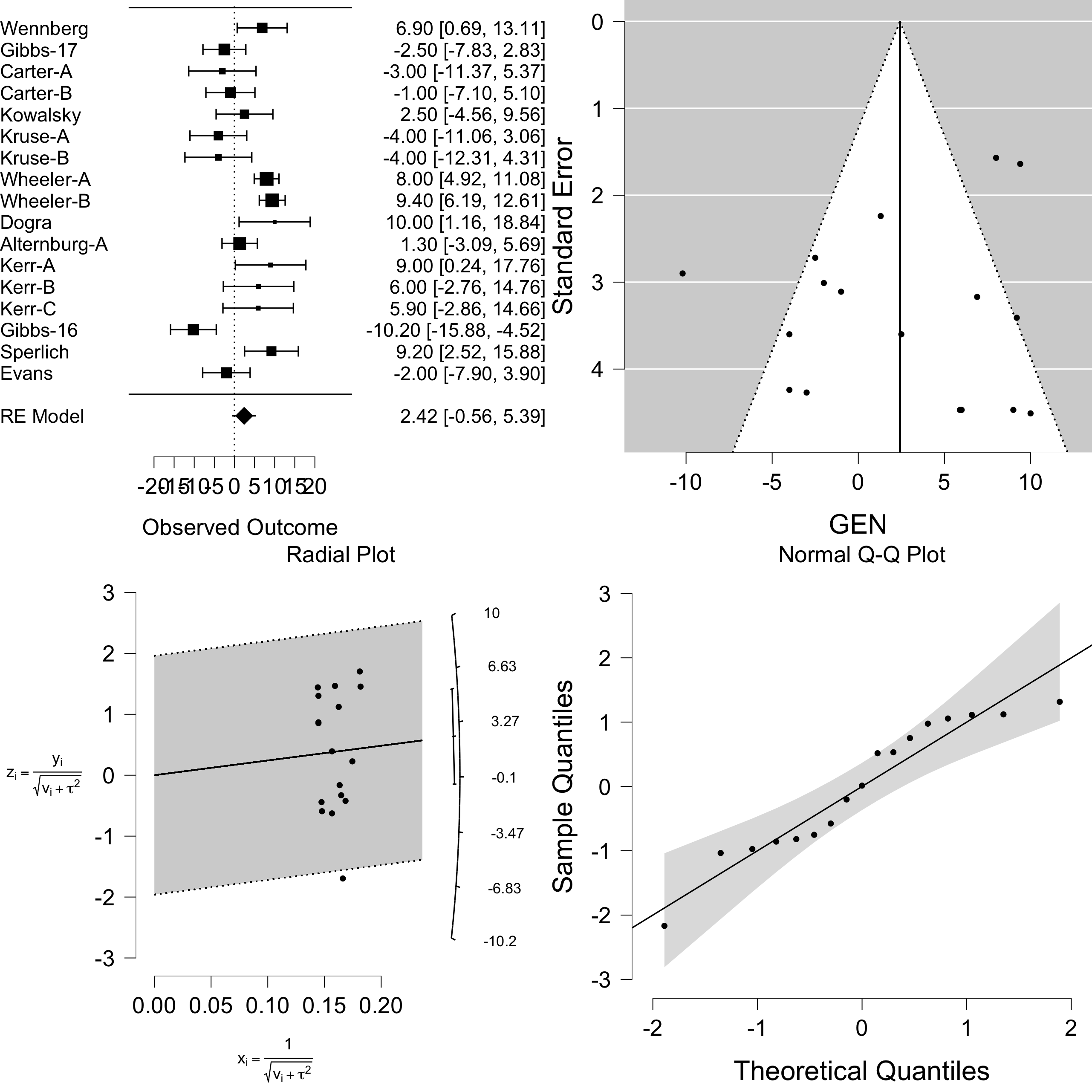


Interpretation: The original funnel plot revealed an overall effect of 2.42. After the trim and fill was performed, the overall effect did not change. Thus, there is no publication bias present for interrupting prolonged sitting when measured with HR.

**
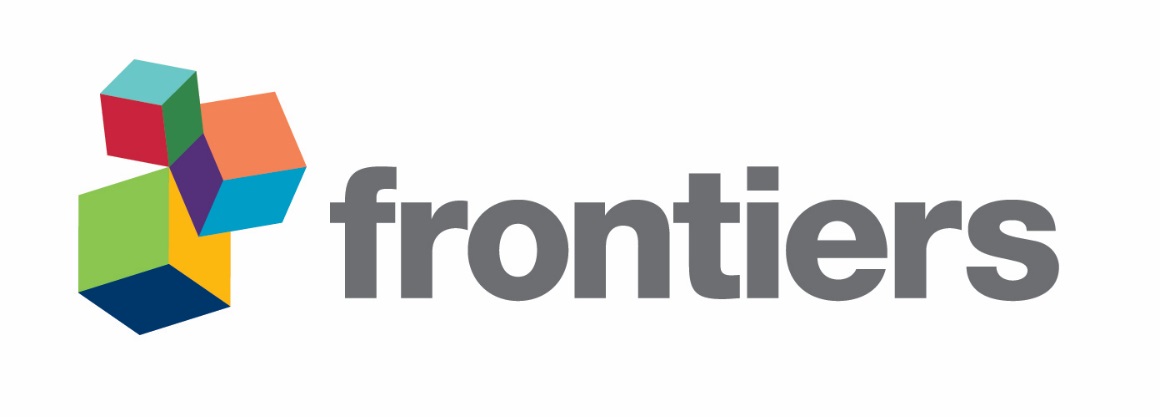
**

**Supplementary Table 1.**

| **Parameter** | **Domain** | **Description** |
| --- | --- | --- |
| **SDNN:** Standard deviation of NN intervals | Time | Combination of SNS an PNS activity describing overall cyclic power of variability |
| **RMSSD:** Root mean square of successive differences | Time | Higher values indicate increased PNS activity measuring differences between successive NN intervals and cardiac vagal activity |
| **HF/LF:** High frequency/low frequency ratio | Frequency | Ratio of HF and LF describing balance between SNS and PNS |
| **HF:** High frequency | Frequency | PNS component (0,15- 0,4 Hz) via respiratory sinus arrhythmia |
| **LF:** Low frequency | Frequency | Combination of SNS/PNS (0,04 – 0,15 Hz) measured via respiratory sinus arrhythmia or blood pressure related change |
| **TV:** Total variance | Frequency | Balance of SNS/PNS displaying overall variance |

Supplementary table 1 displays details regarding the different heart rate variability parameters discussed in the manuscript.
